# Preparation of Microporous Molding Activated Carbon Derived from Bamboo Pyrolysis Gasification Byproducts for Toluene Gas Adsorption

**DOI:** 10.3390/ma16155236

**Published:** 2023-07-26

**Authors:** Yali Wang, Ruting Xu, Mingzhe Ma, Kang Sun, Jianchun Jiang, Hao Sun, Shicai Liu, Yanren Jin, Ting Zhao

**Affiliations:** 1 Institute of Chemical Industry of Forest Products, Chinese Academy of Forestry, Nanjing 210042, China; 15031538625@163.com (Y.W.); xuruting2018@163.com (R.X.); 18951642274@163.com (M.M.); sunkang0226@163.com (K.S.); bio-energy@163.com (J.J.); 2 National Engineering Laboratory for Biomass Chemical Utilization, Nanjing 210042, China; 3 Key Laboratory of Chemical Engineering of Forest Products, National Forestry and Grassland Administration, Nanjing 210042, China; 4 Key Laboratory of Biomass Energy and Material, Nanjing 210042, China; 5 Jiangsu Co-Innovation Center of Efficient Processing and Utilization of Forest Resources, Nanjing Forestry University, Nanjing 210037, China; 6 Shanxi Xinhua Chemical Defense Equipment Research Institute Co., Ltd., Taiyuan 030008, China; jin.yr@163.com (Y.J.); zt870319@163.com (T.Z.)

**Keywords:** molding activated carbon, bamboo-derived tar, bamboo charcoal, gas adsorption, micropore

## Abstract

The effective utilization of charcoal and tar byproducts is a challenge for pyrolysis gasification of bamboo. Herein, the bamboo tar was modified via polymerization and acted as a new adhesive for the preparation of excellent bamboo-charcoal-derived molding activated carbon (MBAC). As compared with pristine tar and other adhesives, the aromatization of tar with phenol increased its molecular weight, oxygenic functional groups, and thermal stability, leading to the decreased blocking impact of charcoal pore and improved bonding and pyrolytic crosslinking effect between charcoal particles. These further contribute to the high mechanical strength, specific surface area, pore volume, and amount of oxygenic functional groups for fabricated MBAC. Owing to the high microporous volume of MBAC, it exhibited 385 mg·g^−1^ toluene and 75.2% tetrachloride gas adsorption performances. Moreover, the pseudo-first-order, pseudo-second-order, and Bangham models were used to evaluate the kinetic data. The toluene adsorption process conforms to the Bangham kinetic model, suggesting that the diffusion mechanism of toluene adsorption mainly followed intraparticle diffusion.

## 1. Introduction

Given the rising environmental concerns associated with the use of fossil fuels, people are constantly searching for innovative ways to reduce greenhouse gas emissions. Biomass is widely regarded as the most promising energy source for reducing greenhouse gas emissions. Due to its unique role as a sustainable carbon carrier, biomass has a significant advantage over other renewable energy sources. As a result, it provides an attractive alternative for meeting energy demands while minimizing environmental impact [1,2]. Forestry residues are recyclable nature resources that can be transformed into useful materials and energy via pyrolysis [3]. The use of forestry waste as a potential source of renewable energy is gaining more and more attention. Among the promising thermochemical conversion routes, pyrolysis gasification of forestry residues has been an essential source of heat [4,5,6,7]. Biomass pyrolysis has been utilized for a considerable period and is generally defined as the thermal decomposition of the biomass organic matrix resulting in liquid bio-oil, solid biochar, and noncondensable gas products [8].

Bamboo is a native plant in many Asian countries, which is fast-growing and can be utilized as a sustainable raw material instead of wood [9,10]. It is well-known that the pyrolysis gasification of bamboo processing residues is an environmentally benign, economic, and carbon negative engineering process [11]. On the one hand, its gas production can be utilized to provide heat and electricity. On the other hand, the bamboo charcoal (BC) byproduct can be easily activated to become bamboo activated carbon (BAC) with high adsorption performances [12,13,14,15].

However, owing to the drawbacks of BAC such as poor mechanical strength and low density, the BAC is hardly applied in the field of gas adsorption, catalysis, etc. The volatile organic compounds (VOCs), such as toluene and benzene, will damage to the liver and nervous system of the human body. The gas adsorption process is an effective method of VOCs pollution purification and is widely used in environment control and life support system of the enclosed space [16]. Another challenge for the pyrolysis gasification of bamboo is the utilization of tar in the liquid byproducts [17]. As bamboo-derived tar (BT) contains polycyclic aromatic hydrocarbons, phenols, as well as other aromatic and nonaromatic organic compounds [18,19], it is difficult to make large-scale and high-value use of this complex BT [20,21,22].

The strength and density of BAC can be improved by using binders [23,24,25,26]. Coal tar pitch with low viscosity contributes to fill the pores, and high char yield is beneficial for enhancing the density of carbon materials during the carbonization stage [27]. Additionally, the highly crosslinked 3D structure of phenolic resin means it follows the solid phase carbonization mechanism [28]. The strong shrinkage of phenolic resin, however, makes it easy for crack flaws to form inside the carbon material, which limits its practical application in the industry. However, the use of coal tar, asphalt, and other binders not only raises the product cost and carbon emissions but also impairs the pore structure and adsorption efficiency of molding bamboo activated carbon (MBAC). In order to replace these fossil-derived binders, Amaya et al. [29] evaluated the char and tar produced by the pyrolysis of Uruguayan eucalyptus wood as raw materials for the preparation of activated carbon pellets. Tomasz et al. [30] analyzed the biomass-derived wood tars as a substitute for commercial coal tar pitch, and their pyrolysis exhibits decreased emissions of polycyclic aromatic hydrocarbons. It is proposed that the wood-derived tar could be utilized as a binder for the preparation of molding activated carbon [31], though its strength and porosity still need to be improved.

Inspired by these, the BT was modified via polymerization with phenol and formaldehyde in this study. The improvement mechanism of modified bamboo-derived tar (MBT) for the prepared columnar MBAC was revealed by comparison with BT, asphalt, and a mixture of asphalt and phenolic resin. The iodine, methylene blue, carbon tetrachloride, and toluene adsorptions of MBACs were performed to investigate the excellent liquid and gas adsorption performances of MBAC prepared from MBT. Moreover, the toluene adsorption of optimized MBAC was simulated to explain the adsorption mechanism of gas molecular.

## 2. Materials and Methods

### 2.1. Materials

BC and BT were collected by Zhejiang Jizhu Biotechnology Co., Ltd. (Huzhou, Zhejiang, China). The moisture, ash, volatile, and fixed carbon contents of BC were 4.94%, 2.94%, 9.58%, and 82.54%, and its carbon (C), hydrogen (H), oxygen (O), and nitrogen (N) contents were 83.97%, 2.11%, 13.33%, and 0.59%, respectively. Phenolic resin and asphalt were purchased from Shanxi Xinhua Chemical Defense Equipment Research Institute Co., Ltd. (Taiyuan, Shanxi, China). Phenol and formaldehyde were purchased from China National Pharmaceutical Group Corp. (Beijing, China).

### 2.2. Preparation of MBAC

A total of 10 g of carboxymethyl cellulose (CMC) was dissolved in deionized water at 80 °C to obtain a 2% CMC solution. For the synthesis of MBT, after the pH pf BT, phenol and, formaldehyde mixture was adjusted to 3–6, the polymerization was consecutively held at 80 °C, 120 °C, and 150 °C for 90 min, 30 min, and 20 min, respectively.

The BC powder (particle size ≤ 0.071 mm), binder (asphalt, phenolic resin, BT, or MBT), and CMC solution were mixed, stirred, and kneaded until the mixture became homogeneous. Then, extruder was formed in the molding machine, and the columnar raw materials were placed in the oven for drying and hardening. Finally, MBAC samples were prepared through the pyrolytic crosslinking and steam activation method. The activated carbon made using asphalt, asphalt and phenolic resin, BT, and MBT as binders were labeled as MBAC1, MBAC2, MBAC3, and MBAC4, respectively. The yield of MBAC during the steam activation was calculated using the following formula:

(1) $$\mathrm{yield}=\frac{m_{2}}{m_{1}}\times 100\%$$

where m_1_ is the mass of resulting char before the activation process and m_2_ is the mass of MBAC.

### 2.3. Characterization

The elemental analysis of the sample (C, H, and N) was characterized using FLASH2000 (Thermo Fisher Scientific, Madison, WI, USA) elemental determination machines, whereas the O content was estimated by difference. Thermal gravimetric (TG) and differential thermal gravimetric (DTG) analysis of different adhesives and MBACs were conducted using a TG209F1 (Netzsch, Selb, Germany) thermogravimetric analyzer under Ar (100 mL/min flow-rate) at a heating rate of 10 °C/min from room temperature to 1000 °C. The molecular weight of BT and MBT were determined using Viscotek model 350 (Malvern Panalytical Ltd., Worcester, GBR, UK) high temperature gel permeation chromatography (HT-GPC). The samples were dissolved in DMF and the injected sample volume was 200.0 μl with 1 mL/min flow rate. The Fourier transform infrared (FT-IR) spectra were analyzed using an IS50 (Thermo Nicolet Corporation, St. Bend, OR, USA) infrared spectrometer from 500 to 4000 cm^−1^ with 32 scans at a resolution of 2 cm^−1^. After MBAC samples were degassed at 150 °C for 10 h under vacuum, their surface physical properties were obtained from the adsorption of N_2_ at 77 K using an ASAP 2460 (Micromeritics, Norcross, GA, USA). The total specific surface area (S_BET_), pore volume (V_total_), micropore volume (V_mic_), and mesopore volume (V_mes_) were calculated according to the Density Functional Theory (DFT) method [32]. Scanning Electron Microscopy (SEM) measurements of samples were carried out using Regulus 8100 (Hitachi, Tokyo, Japan). The SEM images were taken in a secondary electron with acceleration voltage equal to 20 kV and 20 pA emission current.

In order to describe the pore structure and liquid adsorption capabilities of MBAC, the adsorption values for iodine and methylene blue of powder MBAC were determined according to the “Test methods of wooden activated carbon” specification in GB/T 12496.10-2015 [33] and GB/T 12496.10-1999 [34], respectively. Owing to the optimum pore diameter of AC, iodine and methylene blue adsorptions were around 1.0–2.7 nm and 1.7–5.0 nm, respectively [35]; the combination of iodine and methylene blue adsorption experiments could effectively describe the micropore and small mesopore of MBACs.

The carbon tetrachloride and toluene gas adsorption of granule MBAC were determined according to GB/T 12496.5-1999 [36] and GB/T 35815-2018 [37] standards, respectively. The mechanical strength of MBACs were determined according to the GB/T 12496.6-1999 [38] standard. The prepared columnar MBACs with 3 mm diameter were higher than 7 mesh. Typically, after the MBACs were struck with a steel ball in the steel cylinder for 5 min, the mass percentages of residual MBACs higher than 7 mesh were mechanical strength (abrasion resistance) of MBAC samples.

## 3. Results and Discussion

### 3.1. Bamboo Forming Mechanism

**Figure 1:**
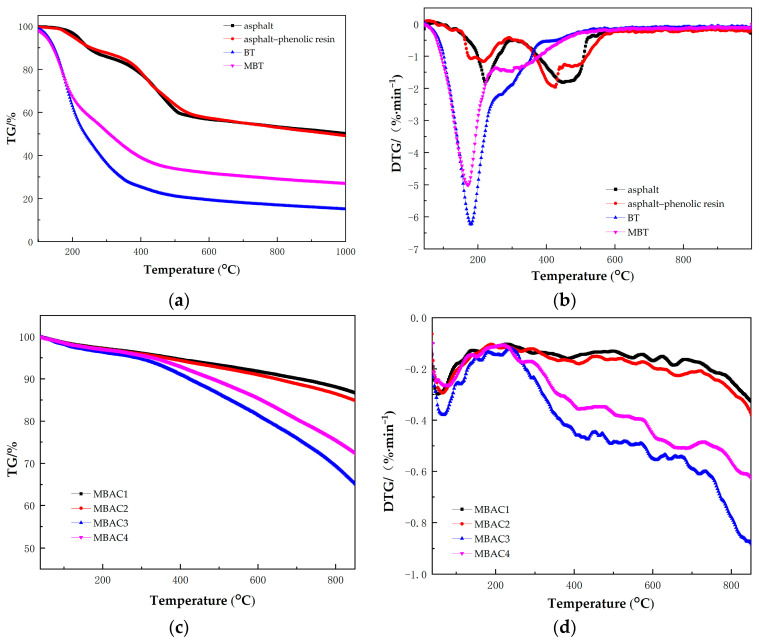
TG (**a**) and DTG (**b**) curves of different binders and TG (**c**) and DTG (**d**) curves of MBACs.

The TG curves of adhesives are displayed in Figure 1a. The TG curves of asphalt and asphalt–phenolic resin are similar. The degradation primarily takes place between 200 °C and 500 °C, and the mass of asphalt and asphalt–phenolic resin remain 50.12% and 49.16% at 1000 °C, respectively. BT shows the lowest thermal stability with only 15.10% remaining at 1000 °C. Its sluggish weight loss starts around 100 °C, while violent weight loss starts around 150 °C, and the rate of weight loss dramatically decreases after 550 °C. The MBT exhibited better thermal stability than BT and similarly severe weight loss at about 150 °C. The pyrolytic crosslinking temperature in this study was set at 550 °C since these four adhesives almost ceased decomposition after that point.

As shown in Figure 1b, the DTG curves show that both BT and MBT have the largest area peaks at about 200 °C, owing to the highest decomposition rate. This may be the volatilization stage of cycloalkane, benzene, phenol, and other low-boiling-point substances [39]. MBT also exhibits a pyrolysis peak at 300 °C. In this stage, there is not only volatilization of substances with high boiling points (e.g., naphthalene and esters substances) but also dehydration reactions of phenol and alcohol and/or carboxyl decomposition of carboxylic acid substances. The asphalt displays clear pyrolysis peaks at 250 °C and 450 °C, while asphalt–phenolic resin exhibits peaks at 200 °C, 400 °C, and 500 °C.

Figure 1c shows the results of the TG curves of MBACs. As illustrated in Figure 1c, the thermal events during the pyrolysis of MBACs can be divided into two stages: The first stage is dewatering and degassing. As the pyrolysis temperature is elevated from room temperature to 300 °C, the moisture, adsorbed volatile organic matter, and volatile nitrogen in MBACs are eliminated [40,41]. The MBACs exhibit a similar weight-loss ratio. In the second stage, the weight loss rates of MBAC3 and MBAC4 are higher than those of MBAC1 and MBAC2 as the temperature is higher than 300 °C. These results suggest that the oxygen-containing functional groups of MBACs are gradually decomposed at and elevated temperature [42], and MBAC3 and MBAC4 present much more oxygenic functional groups than MBAC1 and MBAC2. The DTG curves (Figure 1d) verify the TG curves of MBACs.

Table 1 shows the results of the elemental analysis and HT-GPC of BT and MBT. The mass fractions of carbon, nitrogen, and hydrogen for the MBT decrease, as well as the ratio of n(H):n(C). These results suggest that the modification process increases the density and the amount of polycyclic aromatic hydrocarbon structures (polycondensation structures of aromatic hydrocarbons) of pristine tar. As displayed in Table 1, the results show that the average molecular weight value of MBT is 3.3-times higher than that of BT, consistent with the findings of the elemental analysis. It is suggested that the formaldehyde, phenol, and BT effectively polymerize together in the modification procedure, leading to the enhancement of the relative concentration of high condensation molecules in the BT.

**Figure 2:**
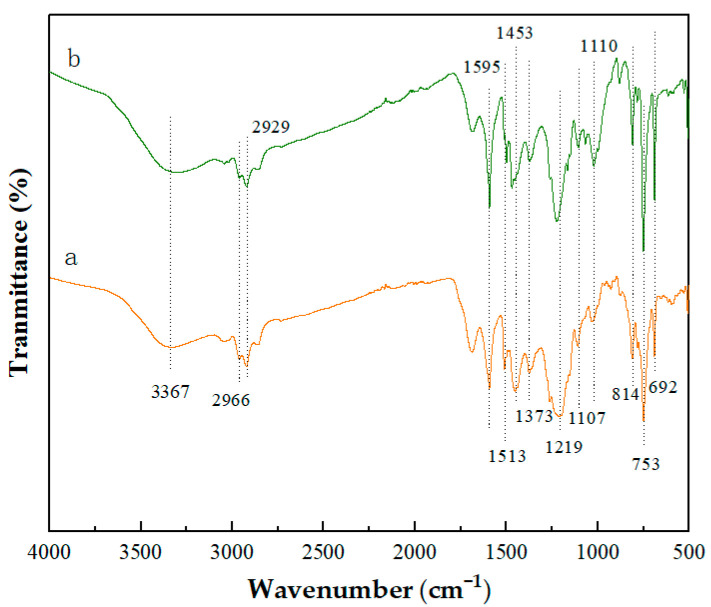
FTIR spectra: (a) BT; (b) MBT.

The infrared spectra of BT and MBT are displayed in Figure 2. It can be seen that the BT exhibits transmission peaks at 3367 cm^−1^ (O-H stretching vibration), 2966 cm^−1^, and 2929 cm^−1^ (asymmetric stretching vibration of saturated aliphatic hydrocarbons -CH_3_ and -CH_2_, respectively), 1513 cm^−1^ and 1593 cm^−1^ (C=C bone vibration of the conjugated double bond of benzene ring), 1453 cm^−1^ (-CH_2_), 1373 cm^−1^ (-CH_3_), 1219 cm^−1^ (asymmetric stretching vibration of aromatic ether bond C-O-C), 1107 cm^−1^ (C-O-C stretching vibration of aliphatic ether bond), and 692–814 cm^−1^ (out-of-plane C-H bending vibration of the aromatic ring substitution). The intensity of aromatic C=C stretching vibrations at 1595 cm^−1^ and C-O-C stretching vibrations at 1100 cm^−1^ in the infrared spectra of MBT was greater than that of BT. It can be presumed that the aromatic compound in the MBT has been increased. This is consistent with the elemental analysis result and shows that the aromatic crosslinking reaction of the BT with phenol and formaldehyde happened during the modification process.

The effects of asphalt, asphalt–phenolic resin, BT, and MBT on the yield, strength, and liquid-phase adsorption properties of MBAC have been studied under the conditions of 550 °C charring temperature, 90 min charring time, 850 °C activation temperature, and 80 min activation time, and the results are listed in Table 2. MBAC2 outperformed MBAC1 in terms of adsorption performance, mechanical strength, and yield, highlighting the advantage of using macromolecular organic substances as the binder to produce MBAC with superior pore space and high strength. The liquid-phase adsorption performance of the generated MBAC synthesized with BT as a binder was marginally better than that of asphalt–phenolic resin, but the strength and yield were much lower. As MBAC was made using the MBT binder, the adsorption performance, strength, and yield were all improved compared with BT.

According to TG analysis, both asphalt and asphalt–phenolic resin show high thermal stability. For the asphalt binder, it can retain a more carbonized structure for crosslinking than BT at 550 °C pyrolysis temperature, leading to increases in adhesion intensity between the BC powder particles. However, the adsorption values for iodine and methylene blue of MBAC1 are only 970 mg·g^−1^ and 195 mg·g^−1^, respectively, though its strength is 92%. During the kneading and high-pressure molding procedures, the asphalt might soften and exhibit liquid properties, resulting in immersing and clogging the pores of BC. Thus, the adsorption performance of MBAC1 is much lower.

Phenolic resin possesses a complex three-dimensional network structure and good thermal stability. As asphalt–phenolic resin is used as an adhesive, it is more difficult for phenolic resin macromolecules to enter into the micropores of BC. The blocking influence of the adhesive on the pores of MBAC2 is weakened, and the bonding effect between BC particles is strengthened. In addition, the high thermal stability can ensure the crosslinking function during the pyrolysis process. Compared with MBAC1, MBAC2 shows higher strength and adsorption performance.

For the BT adhesive, its majority of carbonized structure will be lost during the pyrolysis crosslinking and activation process, leading to decreasing pore-block of BC, releasing the pore structure of activated carbon, and improving the adsorption performance of MBAC3. However, owing to the less remained carbonized structure for crosslinking between BC particles, the MBAC3 exhibits lower strength of 88%. After the modification of BT, both the molecular diameter and thermal stability of MBT are higher than BT according to the TG and GPC analysis. It is difficult for MBT binder to penetrate into micropores of BC. Moreover, the elemental analyses reveal that MBT exhibits more oxygenic functional groups. Therefore, the MBT surrounding the external surface of BC improves the bond and pyrolysis crosslinking between BC particles, and decreases the pore-block of BC. The modification of BT is responsible for the comprehensive improvement of strength, yield, and adsorption performance for the MBAC4.

### 3.2. Optimization of the Preparation Process

As the amount of adhesive has a significant effect on the performance of MBAC, the effects of 8 g, 12 g, and 16 g of MBT on the performance of MBAC were investigated under the conditions of carbonization at 550 °C for 90 min and activation at 850 °C for 80 min. As listed in Table 3, it can be seen that the strength of MBAC increases with the amount of MBT increasing, while both the iodine adsorption value and the methylene blue adsorption value increase first and then decrease. The excess MBT binder may wrap the carbon powder, leading to excessive pore blockage of MBAC and a reduction in MBAC adsorption performance. The iodine adsorption value and methylene blue adsorption value of MBAC reached the maximum value of 1232 mg·g^−1^ and 240 mg·g^−1^, respectively, with an intensity of 91% and a yield of 48.5% when the suitable addition amount of MBT was 12 g.

The effects of activation temperature on MBAC performance were investigated. The amount of MBT was 12 g, carbonization at 550 °C for 90 min, and the activation time was 80 min. As displayed in Table 3, it is obvious that the strength and yield decrease gradually with the increase in activation temperature, while both the iodine adsorption value and methylene blue adsorption values of MBAC initially increase and subsequently decrease. This is due to the fact that raising the activation temperature can hasten the activated reaction rate of water vapor, generate a large number of micropores and mesopores, and enhance the adsorptive property of MBAC. However, if the activation temperature is too high, the yield and intensity of MBAC will be significantly decreased. The iodine adsorption value and methylene blue adsorption value were 1232 mg·g^−1^ and 240 mg·g^−1^, respectively, with an intensity of 91% and a yield of 49.5% as the perfect activation temperature was 850 °C.

As the pore structure of AC is influenced by the activation time [43], the effects of activation time on the performance of MBAC were studied. The amount of MBT was 12 g, carbonization at 550 °C for 90 min, and the activation temperature was 850 °C. The results are shown in Table 3. The strength and yield of MBAC drop gradually with activation time increasing, while the iodine adsorption value and methylene blue adsorption value increase. This is owing to the fact that the stream activation is carried out in the order of pore-creating and then pore-widening. A large number of micropores are formed inside the carbonized product, and the micropores between the carbon microcrystals are generated, resulting in the enhancement of adsorption performance for MBAC. However, when the activation time is prolonged, the micropores widen and even collapsed, and the carbon ablation rate is accelerated, leading to the reduction in strength and yield of MBAC. The MBAC4 exhibited a 1240 mg·g^−1^ iodine and a 247.5 mg·g^−1^ methylene blue adsorption capacity when the ideal activation time was 80 min.

### 3.3. Pore and Surface Structure Characterization

**Figure 3:**
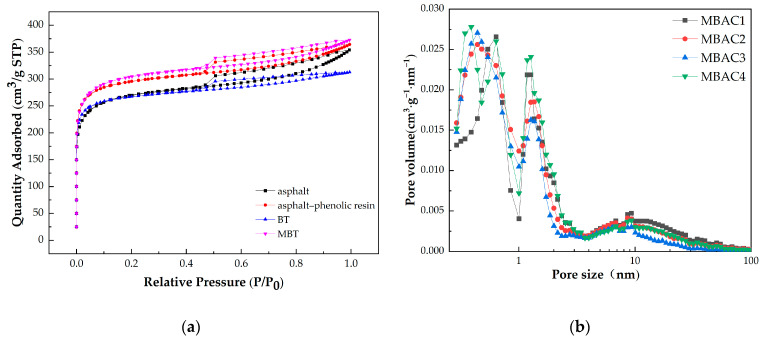
N_2_ adsorption–desorption isotherms (**a**) and pore size distribution (**b**) of MBACs.

The key characteristics of carbonaceous adsorbents are the pore size distribution and surface area because they significantly affect the adsorption capacity or adsorption characteristics [44]. For MBAC produced from different adhesives mentioned in Section 3.1, pore structure and dynamic adsorption performance analyses of toluene and carbon tetrachloride gases were performed. The adsorption–desorption isotherms in Figure 3a are all categorized as type I isotherms. The nitrogen adsorption capacity dramatically increased and single molecule adsorption took place in the low-pressure section (P/P_0_ < 0.1), demonstrating the presence of major micropores in the samples. After P/P_0_ > 0.4, there is a little hysteresis phenomenon in the adsorption curve: the higher the relative pressure, the slower the increase in adsorption volume. This typical H4 hysteresis loop of the MBAC indicates the presence of a slit mesopore. The volume of N_2_ adsorption of MBAC4 is higher than that of MBAC1, MBAC2, and MBAC3 at the same relative pressure, demonstrating the higher specific surface of MBAC prepared by MBT. The specific surface areas of MBAC1, MBAC2, MBAC3, and MBAC4 are 756 m^2^·g^−1^, 893 m^2^·g^−1^, 843 m^2^·g^−1^, and 940 m^2^·g^−1^, respectively.

As shown in Figure 3b, DFT was used to determine the pore-size distribution of the MBAC. Table 4 lists the pore structure, toluene adsorption rate, and carbon tetrachloride adsorption rate of four MBACs. It can be seen that the pore size distributions of the MBAC samples are below 10 nm and mainly microporous. Their micropore volumes are 0.326 m^3^·g^−1^, 0.366 m^3^·g^−1^, 0.334 m^3^·g^−1^, and 0.377 m^3^·g^−1^, respectively. The MBAC4 exhibits the highest total pore volume and micropore volume. These verify the superiority of using MBT adhesive to prepare MBAC. The iodine and methylene blue adsorption values of MBACs generally increase as their total pore volume increase. However, iodine and methylene blue adsorption values of MBAC3 are higher than those of MBAC2, despiteMBAC3’s lower total pore volume and micropore volume. Combining the findings of TG, elemental analysis, and FTIR, these results suggest that the MBAC3 prepared from BT with abundant oxygenic functional groups may exhibit high chemisorption of polar molecules in the liquid phase.

**Figure 4:**
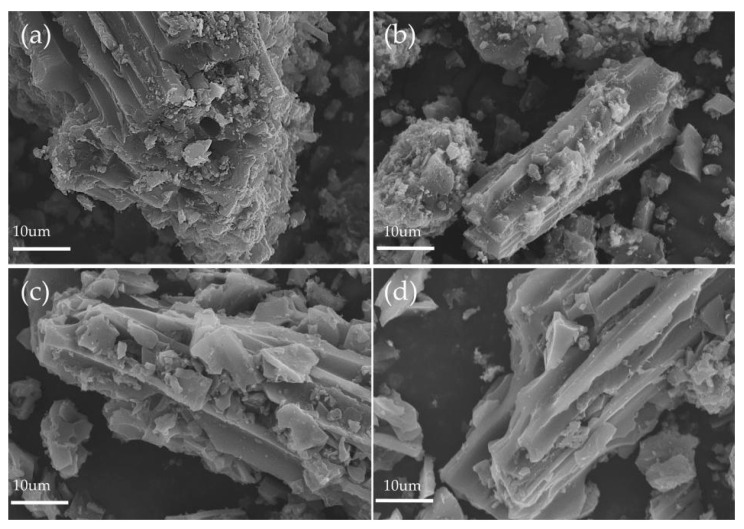
SEM micrographs: (**a**) asphalt; (**b**) asphalt–phenolic resin; (**c**) BT; and (**d**) MBT.

Figure 4 depicts the surface morphology of MBAC produced with different adhesives. The surface of MBAC3 and MBAC4 is smoother than that of MBAC1 and MBAC2, owing to less residual binder after pyrolysis and stream activation, which is in agreement with TG results. All MBACs retain the vessel structure of bamboo that might promote the dispersion of molecules and enhance the adsorption capacity of MBACs.

According to Table 4, the variations in toluene and carbon tetrachloride adsorption rate are similar to the iodine and methylene blue adsorption values. It is distinctly revealed that the relationship between the microporous volume of MBACs and their adsorption performances of toluene and carbon tetrachloride is a positive linear correlation [45]. For the weak polar toluene and nonpolar carbon tetrachloride gas molecule, MBAC materials exhibit mainly pore diffusion confinement and weak chemisorption [46,47]. For the adsorption of toluene molecular, the possible chemisorption mechanism is the interactions of oxygen functional groups of MBACs with an aromatic ring of toluene [48].

### 3.4. Adsorption Kinetics

In order to further explain the adsorption process of weak polar toluene for MBAC, the data were fitted using kinetic models to investigate the adsorption mechanism. Fitting and analyses of kinetic data were carried out using a pseudo-first-order equation and pseudo-second-order equation. The pseudo-first-order equation is used to describe the physical adsorption process. The arrival of adsorbent from solution to adsorbent surface is controlled by a diffusion step [25], and there is only one binding site on the adsorbent surface. The pseudo-second-order equation is mainly used to describe the physical and complex chemisorption process [12]. There are two binding sites on the surface of the adsorbent controlled by the chemisorption mechanism. The nonlinear form of the equation of the pseudo-first and pseudo-second order dynamical model is given as follows [49]:

(2) $$\ln\left(q_{e}{-q}_{t}\right)=\ln q_{e}-k_{1}t,$$

(3) $$\frac{t}{q_{t}}=\frac{1}{k_{2}q_{e}^{2}}+\frac{t}{q_{e}},$$

where q_e_ (g·g^−1^) is the amount of adsorbed dye at equilibrium and q_t_ (g·g^−1^) is toluene adsorption rates at time t. k_1_ (min^−1^) is the rate constant of the pseudo-first-order model. k_2_ (g·g^−1^∙min^−1^) is the rate constant for the pseudo-second-order model.

Table 5 provides the results of the kinetic adsorption model simulations of the MBAC samples for toluene adsorption. Due to that, the R^2^ for the pseudo-first and pseudo-second order kinetic models are between 0.9385 and 0.9860, these equations are not suitable for this adsorption system.

The Bangham model was also used to further examine the kinetic data of toluene adsorption, which is expressed as follows [50,51]:

(4) $$q_{t}=q_{e}\left(1-e^{-k_{3}t^{z}}\right),$$

where z is a constant and k_3_ (min^−z^) is the adsorption rate of the Bangham model.

**Figure 5:**
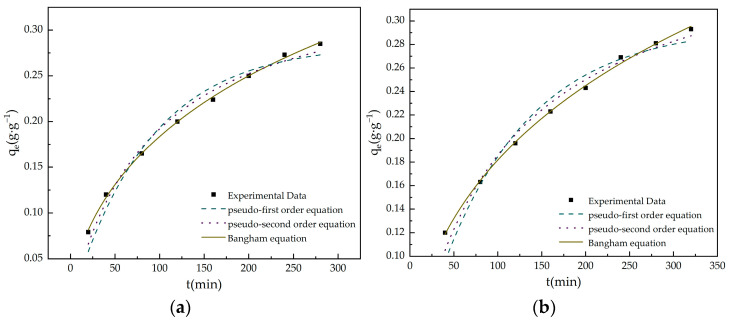

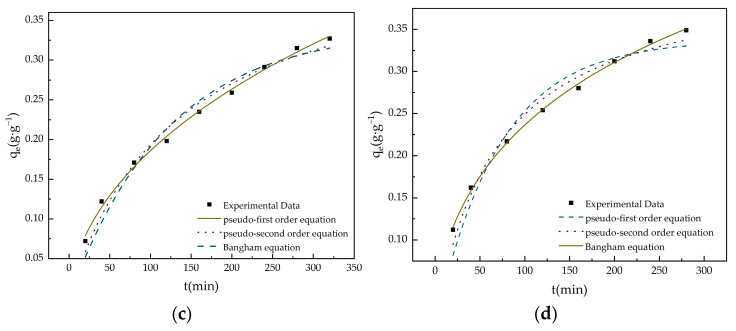
Pseudo-first order, pseudo-first order, and Bangham model fitting curves: (**a**) MBAC1; (**b**) MBAC2; (**c**) MBAC3; (**d**) MBAC4.

The parameters and the R^2^ are presented in Table 5. The Bangham model well fits the experimental data over the entire course with high R^2^ (>0.99) and the experimental adsorption capacities are comparable to the theoretical values predicted by the model (Figure 5). The simulation results suggest that toluene pore diffusion was a major factor in the toluene adsorption diffusion process, consistent with the relationship between the microporous volume of MBACs and their adsorption performances of toluene.

## 4. Conclusions

This study shows that the aromatized BT can be effectively used as an adhesive for the preparation of MBAC. Owing to the high average molecular weight, stability, and concentration of oxygenic functional groups of MBT after modification, its improved bonding and pyrolytic bridging effects for the BC particles and reduced blocking of BC micropore result in the increased micropore structure and mechanical strength of MBAC. The micropore volume of MBAC is positively responsible for its adsorption performances of toluene and carbon tetrachloride gas molecular. According to the kinetic fitting, the adsorption of toluene on MBAC is consistent with the Bangham kinetic model, and its adsorption process includes surface adsorption and mainly pore diffusion.

## Figures and Tables

**Table 1:** Elemental analyses of coke tar.

Sample	Elemental Analysis				n(H):n(C)	HT-GPC
	C (%)	N (%)	H (%)	O (%)		Average Molecular Weights
BT	77.25	1.49	6.77	14.49	1.052	2304
MBT	74.73	1.08	6.21	17.98	0.997	7716

**Table 2:** Effects of binders on the properties of MBAC.

Samples	Iodine Adsorption Value (mg·g^−1^)	Methylene Blue Adsorption Value (mg·g^−1^)	Mechanical Strength (%)	Yield (%)
MBAC1	970	195	92	53.4
MBAC2	1092	202.5	95	56.7
MBAC3	1181	225	88	46.0
MBAC4	1232	240	91	48.5

**Table 3:** Effects of preparation conditions on the properties of MBAC4.

Preparation Conditions		Iodine Adsorption Value (mg·g^−1^)	Methylene Blue Adsorption Value (mg·g^−1^)	Mechanical Strength (%)	Yield (%)
The additional amount of modified tar ^1^	8g	1189	210	84	49.6
	12 g	1232	240	91	48.5
	16 g	1044	195	93	50.6
Activation temperature ^2^	800 °C	1067	195	92	59.2
	850 °C	1232	240	91	48.5
	900 °C	1134	217.5	81	25.6
Activation time ^3^	60 min	1007	187.5	94	55.7
	80 min	1232	240	91	48.5
	100 min	1240	247.5	89	40.1

^1^ Preparation condition: carbonization temperature: 550 °C, carbonization time: 90 min, activated temperature: 850 °C, activated time: 80 min. ^2^ Preparation condition: MBT addition: 12 g, carbonization temperature: 550 °C, carbonization time: 90 min, activated time: 80 min. ^3^ Preparation condition: MBT addition: 12 g, carbonization temperature: 550 °C, carbonization time: 90 min, activated temperature: 850 °C.

**Table 4:** Pore-structure parameters, toluene adsorption activity, and carbon tetrachloride activity of MBACs.

Samples	Specific Surface Area (m^2^·g^−1^)	Pore Volume (m^3^·g^−1^)				Average Pore Size (nm)	Toluene (mg·g^−1^)	Carbon Tetrachloride (%)
		Total	Micro	Meso	Micro Ratio (%)			
MBAC1	756	0.449	0.326	0.118	72.73	2.05	257	47.9
MBAC2	893	0.466	0.366	0.096	78.54	1.96	354	55.6
MBAC3	843	0.402	0.334	0.066	83.08	1.85	273	48.8
MBAC4	940	0.478	0.377	0.099	78.81	1.94	385	75.2

**Table 5:** Fitting parameters of kinetics equations for the toluene adsorption on MBACs.

Samples	q_e_^exp^ (g·g^−1^)	Pseudo-First Order Kinetic Model			Pseudo-Second Order Kinetic Model			Bangham Model		
		q_e_ (g·g^−1^)	k_1_ (min^−1^)	R^2^	q_e_ (g·g^−1^)	k_2_ (g·g^−1^·min^−1^)	R^2^	q_e_ (g·g^−1^)	k_3_ (min^-z^)	R^2^
MBAC1	0.285	0.272	0.0113	0.9600	0.276	0.0322	0.9857	0.287	0.028	0.9985
MBAC2	0.293	0.276	0.0098	0.9534	0.277	0.0248	0.9795	0.295	0.029	0.9978
MBAC3	0.327	0.314	0.0083	0.9619	0.318	0.0168	0.9784	0.318	0.014	0.9950
MBAC4	0.349	0.336	0.0139	0.9385	0.359	0.0345	0.9760	0.367	0.040	0.9985

## Data Availability

The data that support the findings of this study are available from the corresponding author upon reasonable request.
